# Mobilizing for Community Benefits to Assess Health and Promote Environmental Justice near the Gordie Howe International Bridge

**DOI:** 10.3390/ijerph17134680

**Published:** 2020-06-29

**Authors:** Natalie Sampson, Simone Sagovac, Amy Schulz, Lauren Fink, Graciela Mentz, Angela Reyes, Kristina Rice, Ricardo de Majo, Cindy Gamboa, Bridget Vial

**Affiliations:** 1 Department of Health & Human Services, University of Michigan-Dearborn, Dearborn, MI 48128, USA; 2 Southwest Detroit Community Benefits Coalition, Detroit, MI 48209, USA; simonesagovac@gmail.com; 3 Health Behavior Health Education, School of Public Health, University of Michigan, Ann Arbor, MI 48109, USA; ajschulz@umich.edu (A.S.); rdemajo@umich.edu (R.d.M.); 4 Detroit Health Department, Detroit, MI 48207, USA; finkl@detroitmi.gov (L.F.); agreyes@dhdc1.org (A.R.); cgamboa@dhdc1.org (C.G.); 5 Department of Anesthesiology, Michigan Medicine, University of Michigan, Ann Arbor, MI 48109, USA; gmentz@med.umich.edu; 6 Center for Global Health Equity, University of Michigan, Ann Arbor, MI 48109, USA; klrice@umich.edu; 7 Michigan Environmental Justice Coalition, Detroit, MI 48209, USA; bvial@umich.edu

**Keywords:** air pollution, community benefits, environmental justice, Health Impact Assessment, goods movement, transportation infrastructure, community-based participatory research

## Abstract

Transportation infrastructure decisions contribute to social, economic, and health inequities in the U.S. Health Impact Assessments (HIAs) may improve understanding of potential strategies to mitigate adverse effects on quality of life from planned developments. We use the Gordie Howe International Bridge (GHIB), currently under construction in southwest Detroit, MI, as a case study to examine 15 years of community mobilization, which resulted in community benefits that included an HIA. We describe community engagement processes, household survey methods, and select findings of the baseline HIA, with a focus on their application to inform recommendations to promote quality of life. Baseline HIA results indicated significantly higher self-reported asthma rates among children living within 500 feet of trucking routes. Residents reported substantial economic (e.g., decreased home values), health (e.g., adverse outcomes, lack of health care access), and environmental (e.g., air pollution) concerns related to the GHIB. We discuss specific recommendations, based on HIA results, to reduce adverse impacts of the GHIB. These recommendations will inform ongoing community benefits negotiations. This case study provides lessons for community, academic, and government partners conducting HIAs, especially during building and operation of major infrastructure, and discusses their potential role in improving community engagement opportunities towards environmental justice.

## 1. Introduction

**Figure 1:**
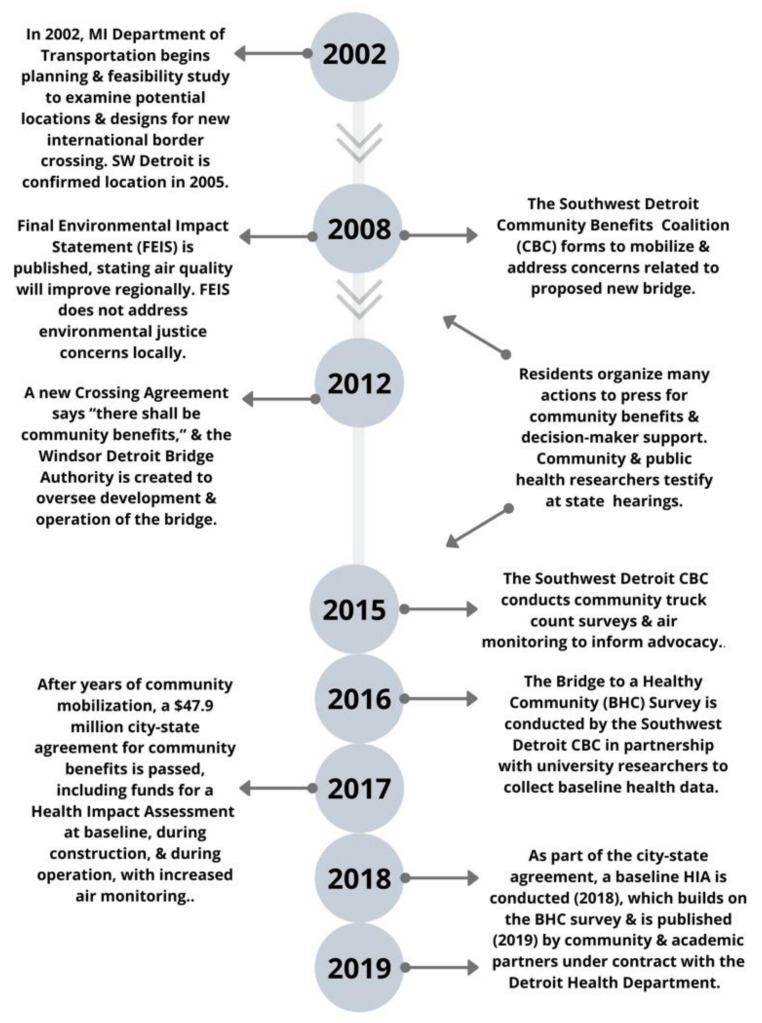
Timeline of key events leading up to the Gordie Howe International Bridge (GHIB) Health Impact Assessments (HIA).

Over USD 122 billion worth of imports and exports traverse internationally each year between Detroit, MI, and Windsor, ON at the busiest land border crossing between Canada and the U.S. [1]. Over 15 years ago, local, state, and federal agencies began discussions about the need for a new bridge to offer direct freeway-to-freeway cross-border connection, and to supplement the privately-owned, aging Ambassador Bridge. As shown in Figure 1, in 2002, the Michigan Department of Transportation (MDOT) initiated a feasibility study [2] to assess the project’s need. While multiple locations were initially identified as potential sites, MDOT formally began a scoping process, narrowing to Detroit’s Delray neighborhood and the surrounding Southwest Detroit region in 2005. In 2008, a 4-year, nearly USD 40 million federal Environmental Impact Statement (EIS) concluded that, despite the doubling of commercial truck traffic in the region, from 10,000 to 20,000 trucks per day, air quality would improve in the region [3]. The regional scale of the EIS did not consider the disproportionate impact of increases in air pollution in areas immediately proximate to the new bridge as it would be built and become operational. Already burdened by cumulative environmental exposures [4,5], nearby residents launched the Southwest Detroit Community Benefits Coalition (CBC) in 2008, to request community benefits to protect the quality of life in the context of the pending bridge.

The bridge became increasingly likely when Canadian officials announced they would provide USD 550 million towards the building of a second bridge border crossing in 2010 and, in 2011, Michigan’s Governor Snyder was elected and announced, in his ‘State of the State’ address, that building the bridge would be a priority for his administration [6]. The Windsor-Detroit Bridge Authority (WDBA) was formed in 2012, to oversee the construction and operation of what became named the Gordie Howe International Bridge (GHIB). Between 2008 and 2014, the community organized to influence decision-makers. In 2014, Detroit City Council granted authority to a GHIB Community Advisory Group (CAG), comprised of city and state agency leaders and local community and business leaders, to represent Delray and Southwest Detroit in defining community benefits. In 2018, construction began on the GHIB.

In this paper, we describe community-driven processes used to advocate for benefits to residents in the GHIB area that resulted in support for a Health Impact Assessment (HIA), among many other outcomes. Emerging at the intersection of public health and community development practices, HIAs are, “An evidence-based tool used to influence decisions on policies, plans, and projects before they are finalized to create more equitable, healthier communities [7].” We describe selected findings from the HIA (see http://bit.ly/BaselineHIA for complete results) [8], conducted prior to construction of the GHIB, and recommendations grounded in HIA results with the goal of reducing adverse economic, health, and environmental impacts for the surrounding community. Our purpose here is to illustrate the interactive process of community mobilization to request that an HIA be conducted, the community-centered process of the HIA, and the subsequent use of findings from that HIA to inform specific recommendations to protect the health of the community from adverse impacts of this new development. 

## 2. Background and Context

### 2.1. Cumulative Impacts for Delray and Surrounding Community

As this paper goes to press, the GHIB is now under construction in an area already disproportionately burdened by environmental exposures [4,5]. Delray and the greater Southwest Detroit region, following national trends, became increasingly industrialized in the twentieth century, with the building of many facilities and a major transportation corridor. In the immediate vicinity of the GHIB location, residents live nearby a US Steel mill and DTE energy plant (both coal-fire powered), a wastewater treatment plant that incinerates sewage for a third of Michigan’s population [9], Marathon oil refinery, and Ford Motor plant, among other industrial sites. A transportation network accompanies this industry, including primary rail lines for the region and a nexus of major freeways. In 1929, the Michigan Central Railroad completed construction of the Ambassador Bridge, which remains among the busiest international border crossings today, located on the Detroit River just a few miles from the GHIB site. In the 1950s and 1960s, MDOT built I-75, the major freight thoroughfare from Ontario in Canada to Florida in the United States. Today, I-75 passes through Southwest Detroit at the Ambassador Bridge heading south towards Ohio. It is part of the once-called ‘NAFTA super-highway’ to the Mexican border and remains an efficient route for the mass movement of goods. 

**Figure 2:**
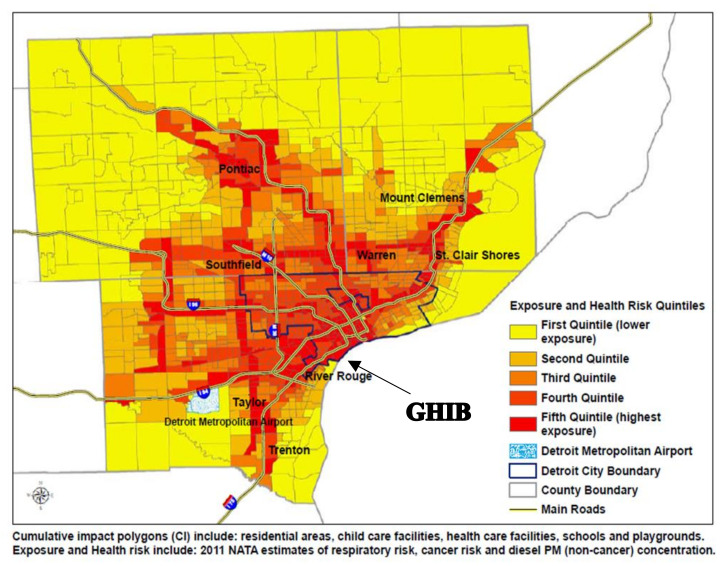
Diesel particulate matter exposure, cancer and respiratory risk attributable to air pollution in the Detroit Metropolitan Area [9].

Given the robust global literature on the near roadway health effects of air pollution [10,11,12,13] and the lived experiences in a heavily polluted community, the CBC and many residents mobilized because they anticipated a worsened quality of life and health disparities associated with bridge-related activities, particularly for those most vulnerable. With extensive transportation-related and industrial air pollution sources, the Delray neighborhood is in a part of Wayne County that is currently in non-attainment of EPA’s standards for sulfur dioxide (since 2013) and ozone (since 2018) [14]. Health impacts and disparities related to air pollution are well documented, and exposure is associated with respiratory, cardiovascular, reproductive, and cognitive outcomes, as well as premature mortality [15]. Living near major transportation infrastructure can also increase noise pollution exposure, with adverse implications for health related to cardiovascular disease and diabetes [16]. Other communities at the fence line of major freight infrastructure have expressed concerns about the many threats to quality of life [13,17,18]. Together, multiple environmental exposures create an additive or cumulative threat to environmental quality and its impacts on human health. For example, Figure 2, created by Community Action to Promote Healthy Environments, illustrates that the GHIB impact area currently experiences cumulative air pollutants and associated health risks that are among the highest in the tri-county (Wayne, Oakland, and Macomb Counties) area [19]. Further, Southwest Detroit is home to many families, and has among the highest proportions of children under the age of five in the Detroit Metropolitan Area [18]. Young children are particularly susceptible to adverse health effects of environmental exposures, such as air pollution, given that they consume more air per unit of body weight and are still developing when compared to adults [20,21,22].

### 2.2. Health Impact Assessment to Inform Community Benefits 

Since 2008, the Southwest Detroit CBC has focused on residents’ concerns about the uncertain impacts of the new bridge infrastructure compounding ongoing industrialization of the area and long-term disinvestment in the Delray neighborhood. Particular concerns revolved around the perceived failure of the 2008 EIS scoping process described above to recognize local environmental justice implications of the GHIB, and its disproportionate adverse impacts on the already overburdened residents of Delray. The EIS did not discuss impacts of the GHIB for those living closest to the bridge, and no mitigation measures were legally required to address potential disproportionate environmental exposures. Thus, the community’s strategy became to pursue community benefits—contracts outside of the EIS process—which would be legally binding to deliver protections from the adverse impacts of the bridge. 

**Figure 3:**
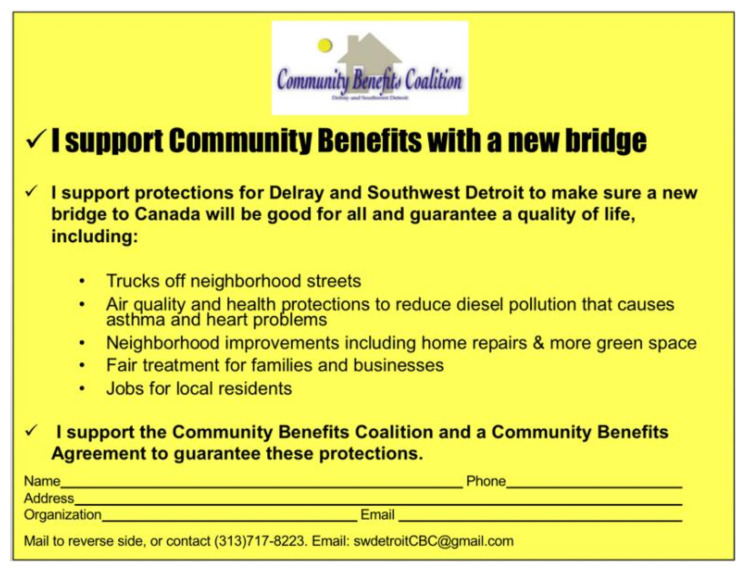
Southwest Detroit Community Benefits Coalition postcard signed by members to express consensus in support for community benefits.

Between 2008 and 2018, the CBC focused on residents’ priorities by:

- Testifying at state hearings at multiple points in the bridge deliberations;
- Organizing a ‘Visit Before you Vote’ campaign, and a tour of the affected area for elected officials and bridge decision-makers to humanize land use decisions;
- Holding call-in days to mayors, city council, and the Governor’s office to inform specific decisions;
- Signing thousands of postcards and organizing petitions to advocate for community benefits (see Figure 3);
- Conducting community-led studies on truck traffic and routes, air quality, and health of residents to share with decision-makers;
- Door-knocking and holding countless CBC meetings to inform residents of data, pending decisions, and opportunities to engage.

As the CBC mobilized, they specifically advocated for an HIA, among their policy requests of decision-makers. Community leaders observed how HIAs had been used globally to assess quality of life in relation to major developments over the last few decades [23,24], with some success addressing community concerns [25]. Community quality of life is multidimensional, with many domains (e.g., employment, education, housing) and an inherently subjective construct, with major implications for overall well-being [26]. Whereas an EIS, traditional risk assessment, or single-source exposure study may be limited [27], HIAs can more comprehensively assess diverse social and health indicators, in addition to specific anticipated adverse impacts of air quality, associated with a planned development such as the GHIB. 

The CBC also educated U.S., Michigan, and Detroit decision-makers on strategies undertaken to address environmental health on the Canadian side of the pending bridge that were not being taken on the U.S. side. For example, local laws in Canada supported development of more elaborate vegetative buffers than those planned in the U.S. to reduce air and noise pollution in the neighborhoods adjacent to the freeway. The CBC conducted international bus tours for elected officials to advocate for the same protections for residents on both sides of the border.

While federal and state government leaders made major decisions about the GHIB, the role of local leaders was also substantial, and many economic and political factors influenced the community mobilization strategies within Detroit. In 2013, the city filed for bankruptcy and was placed under state emergency management. The Detroit Health Department (DHD) was largely dismantled and privatized, with extremely limited staff and services remaining. That same year, the City Council shifted from all at-large members to seven district-specific and two at-large. Emerging from bankruptcy and ending emergency management in 2014, City Council and the mayor regained authority over the city’s budget. At this time, the state proposed buying city-owned vacant land needed for the bridge. Residents organized a counter proposal to allocate revenues from the land sale toward community benefits and to establish the sanctioned CAG. Overcoming opposition, the community won a precedent-setting allocation of USD 750,000 to be used in Delray to remove more than 60 dangerous vacant structures from around the Delray children’s recreation center—a long-unaddressed community-identified need aligning with the mayor’s priority of blight removal. Detroit’s mayor, administration and City Council continued to influence deliberations over community benefits by working with the community during a second sale of city-owned land and transfer of roads for the project area. Ongoing events influenced the CBC’s strategic actions, and who they engaged locally in their quest for benefits.

In 2017, following sustained community mobilization and through negotiations with the Windsor Detroit Bridge Authority, USD 47.9 million were committed in an agreement between the City of Detroit and the State of Michigan (henceforth, the City-State Agreement) for community benefits in SW Detroit, specifically, to address human impacts and maximize benefits such as jobs associated with construction and operation of the GHIB. Funds were designated to support: (1) the monitoring of health impacts of bridge construction and operations through a three-phase Health Impact Assessment (HIA), with increased monitoring of air quality over time; (2) a Neighborhood Fund, which would later be used to support an optional “home swap” initiative that allows residents of designated areas near the bridge to trade their home for another, owned by the Detroit Land Bank Authority elsewhere in Detroit; and (3) a home mitigation program for windows and air filtration to offset impacts during future bridge operation. Of the USD 47.9 million, USD 2.4 million were committed to the HIA and air quality monitoring managed by the City’s Health Department.

**Figure 4:**
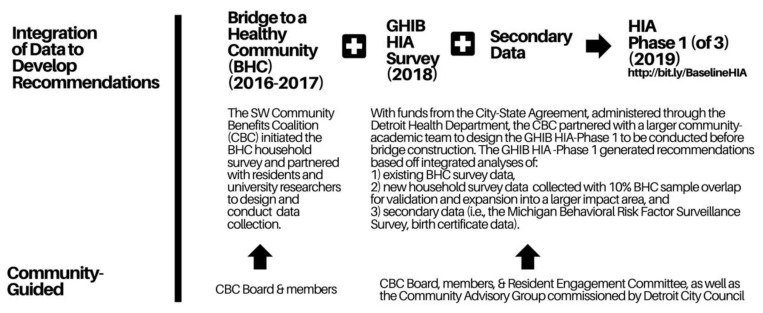
The GHIB Health Impact Assessment—Phase 1: Integration of Data to Develop Community-Guided Recommendations.

Below, we describe community-initiated surveys, supplemented with secondary data, to assess the community’s baseline health before the bridge construction and to inform recommendations to mitigate the potential adverse health impacts of the GHIB. This process is depicted in Figure 4.

## 3. Materials and Methods 

### 3.1. Community-Led Data Collection

In 2015, well before the 2017 announcement of the City-State Agreement, the CBC administered a brief Quality of Life survey to participants in community meetings. As community discussions continued to focus on health concerns, the CBC sought a partnership with public health researchers with expertise in survey research at the University of Michigan (UM) Dearborn. This partnership led to an initial health survey of residents in the bridge impact area. The CBC, guided by its community-elected board, conducted the initial health survey in 2016 and 2017 (Bridge to a Healthy Community (BHC), described below). Draft surveys were presented to community stakeholders for input, including CBC board members, and local bilingual residents, who were hired as interviewers. Through practice sessions to pretest the survey, the interviewer team finalized the initial survey tool. Survey participation was promoted through door-to-door information, phone-calls, and at community meetings. In April 2017, preliminary findings from the BHC survey and a general overview of the literature on near roadway health impacts were presented at two meetings organized by the CBC: (1) for the staff of Michigan Governor Snyder at UM School of Public Health; and (2) at a community meeting at the Community Health and Social Services Center. 

**Figure 5:**
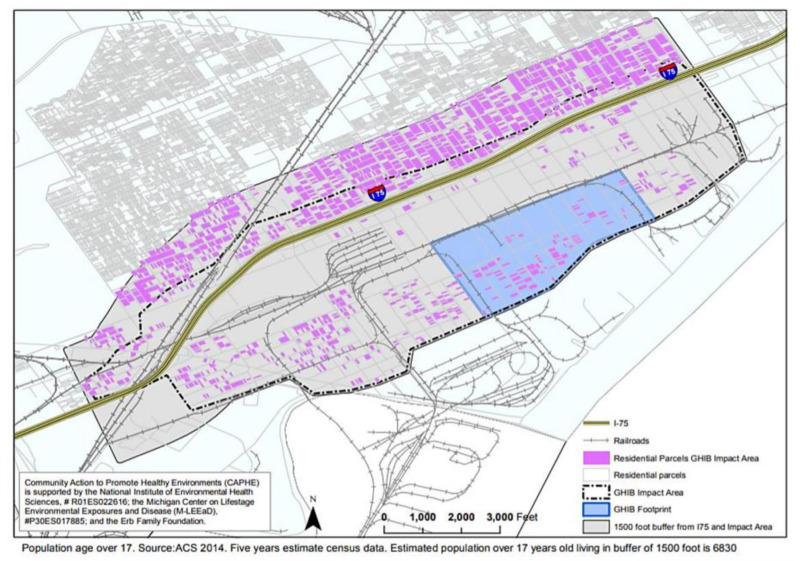
Gordie Howe International Bridge Survey area: Residential parcels in impact area and 1500-foot buffer.

**Figure 6:**
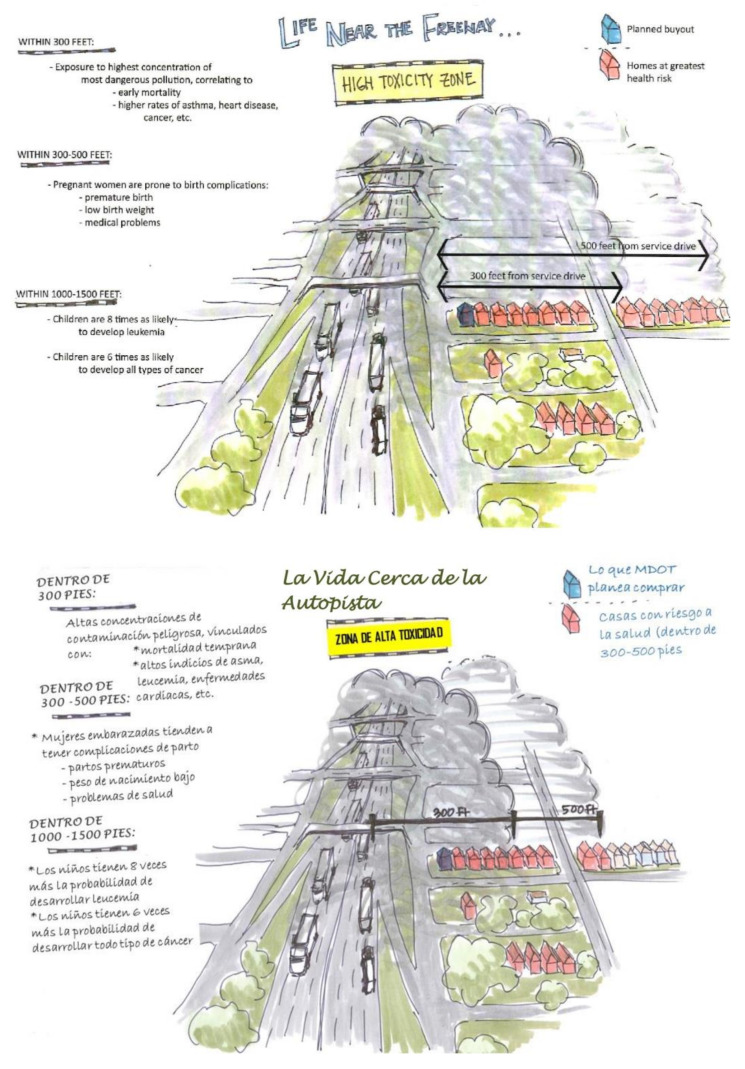
Life near the Freeway Illustration Shared with Survey Participants, English and Spanish (Artist Credit: Sandra Yu Stahl, 2015).

The UM Dearborn Institutional Review Board (IRB) approved the BHC survey and data collection processes (2016). All survey team members participated in a certification process during which they learned about research ethics and survey protocols and practiced asking closed- and open-ended questions. The 100-item bilingual survey was administered face-to-face using Qualtrics software on handheld tablets. The survey took approximately 45-min to complete, and each respondent received USD 5 cash appreciation for their time and contributions. All households in the study area, shown within the dotted line in Figure 5, were invited to participate. Respondents in 302 households, roughly one in three eligible households, completed the survey, exceeding a 25% target response. Due to elaborate messaging about the survey at community meetings and door-to-door distribution of flyers, many residents anticipated the survey team. Interviewers visited each household at least 3 times, at varying times of day and week. The survey team met regularly to discuss survey management, to ensure a representative response across the sampling frame. Survey interviewers requested to speak with the head of household and only interviewed respondents 18 years of age or older. Participants were asked questions related to demographics, insurance status, potential environmental exposures (e.g., occupational), their own health conditions, perceptions of their neighborhood, and recommendations for decisionmakers, as well as age, insurance status, tobacco use, and health conditions for all others in their household. Placed near the end of the survey, to prevent response bias, participants were presented with the scientifically-informed image in Figure 6, to provide a context for the final questions regarding the likelihood of relocation and interest in a series of potential interventions (e.g., air filters) or programs (e.g., home swap). The 2016–2017 BHC survey tool and process provided the foundation for the 2018 HIA.

### 3.2. Designing a Community-Driven Health Impact Assessment 

During the 2017 negotiations for community benefits programs, community support for further health protections continued, and an HIA with three phases—assessing community health before, during, and after bridge construction—was funded as part of the City-State Agreement. 

**Figure 7:**
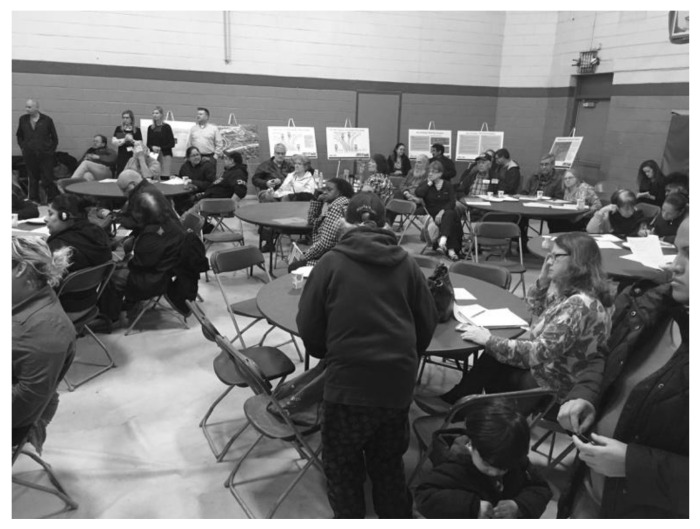
The Health Impact Assessment process built on existing mobilization efforts of the SW Detroit Community Benefits Coalition (CBC), and entailed much input from CBC members and the Resident Engagement Committee, as well as the GHIB Community Advisory Group. The HIA team attended several community meetings organized by the CBC to present on the proposed methods and preliminary findings, for example.

The DHD specified community input at designated points in the request for proposals for the 2018 HIA survey: developing content, piloting, and discussion and interpretation of preliminary results. The team contracted to conduct the HIA included: the Detroit Hispanic Development Corporation (DHDC) (as fiduciary), the CBC, UM School of Public Health (SPH), and UM Dearborn. DHDC and the CBC are both members of the GHIB CAG. The CBC was responsible for organizing community input opportunities and recruiting community interviewers in meetings, such as that depicted in Figure 7. UM SPH and UM Dearborn were responsible for providing survey and analysis expertise. All team members partnered in the overall design and met regularly with community groups to assure input. Specifically, the HIA team met with the CAG in June 2018, to present and discuss the initial findings from the 2016–2017 BHC survey, followed by opportunities for the community to suggest new or modified content for the 2018 GHIB survey. In July 2018, a draft of the revised survey was pilot tested with community residents, who provided feedback and additional input into the survey tool, prior to finalization. The CAG was apprised of progress over the summer and early fall, while the survey was in the field. Preliminary survey results were presented at meetings of the CBC’s Resident Engagement Committee in November 2018 and of the CAG in December 2018 and February 2019. These CAG meetings specifically focused on survey results for the baseline health status of residents of the GHIB area and discussion of potential scientifically-informed recommendations for strategies to minimize potential adverse health impacts of bridge construction and operations on health.

The GHIB survey consisted of a two-stage, stratified random sample of residents living in the area surrounding the GHIB project footprint. As shown in Figure 5, the survey area included the area immediately surrounding the footprint of the bridge and encompassed residents of the area up to 1500 feet north of the new I-75 service drive. This distance was selected based on the literature on near-roadway health effects and commonly known impacts on quality of life within the community, and to provide enough distance for comparative analyses. The survey area included approximately 2586 parcels, excluding 96 parcels in the bridge footprint, where all residences were bought out through eminent domain purchases in 2016. Based on data from the American Community Survey (ACS), an estimated 75% (*n* = 1943) of those households were occupied in 2014—approximately 74% in the impact area and 76% in the area extending 1000 feet north of the impact area. In the first stage of sample selection, census blocks were selected with probabilities proportional to size, using the most recent data available regarding counts of households at the census block level.

In the second stage, a sample of households was selected in each selected census block, with probabilities accounting for household type (e.g., single family, multi-family). A probability sampling method ensures that all population members have a known, non-zero probability of selection. To address the issue of geospatial and temporal confounding (e.g., that the surveys conducted in 2016–2017 (BHC) and in 2018 (GHIB) covered two different areas, making it difficult to disentangle differences related to geographic area from those due to temporal changes), the GHIB survey sample was drawn as a cross-sectional probability sample with an approximately 10% overlap with respondents from the BHC survey.

The two surveys (BHC 2016–2017 and GHIB 2018) can be treated as two cross-sectional samples done in different spatial areas at two time points with 10% overlap. Based on experience of the BHC survey team, the GHIB survey sample for the new or buffer area was created estimating a 33% response rate. In the impact area, where GHIB interviewers would be returning to housing units that had previously participated in the BHC survey, a 50% response rate was estimated. For the new or buffer area, extending 500–1500 feet north of I-75, we sought to complete at least 100 surveys in an area with 478 housing units. Since this area was slightly farther from the GHIB footprint, residents had received less information about the development and the CBC. Thus, we drew random samples using a 1:3 sampling fraction for the new or buffer survey area (from 500–1500 feet north of I-75) and using a 1:2 sampling fraction in the previously-sampled impact area. 

To maximize comparability and integration of data across the BHC and GHIB surveys for the baseline GHIB HIA, survey questionnaires were kept as consistent as possible. Limited modifications were made to be responsive to evolving community concerns and the HIA process, as well as to improve the survey’s rigor, for example, to include: more response options in some instances; questions regarding pregnancy, birth outcomes, and the use of supplemental oxygen; and several items related to housing renovations or features (e.g., heating sources, air conditioning use). 

Secondary data supplemented the survey data. Birth certificate data for the 48209 zip code, which encompasses the whole study area and are available from the Michigan Department of Health and Human Services Vital Statistics Division, were used to complement self-reported health data obtained through the BHC and GHIB surveys. Data from Michigan Behavioral Risk Factor Surveillance Survey (MiBRFSS) were used to supplement survey data, and, in particular, to examine asthma prevalence in Detroit and the state of Michigan. The asthma item from the MiBRFSS asks whether individuals have been told by a health care provider that they have asthma, which differs from the BHC and GHIB surveys, which simply ask if each individual has asthma. The MiBRFSS data reported is aggregated from 2014–2016.

### 3.3. Data Analysis

Data from the 2016–2018 surveys were cleaned using standard procedures and validated, as described in further detail in the HIA report available online (http://bit.ly/BaselineHIA). Among the 146 surveys completed during the 2018 field period, 27 replicate interviews were conducted at households with the respondents who had completed the BHC interview in 2016–2017. We used Pearson’s correlation coefficients to assess consistency of responses to 51 survey items (e.g., gender, smoking status, length of residence in Detroit, employments status) by the same respondent at the two points in time. The following standard characterization was used to evaluate these results: r < 0.4 ‘Not consistent’; 0.4 ≤ r < 0.6 ‘Poor consistency’; 0.6 ≤ r < 0.8 ‘Good consistency’; 0.8 ≤ r ‘Excellent consistency.’ Using this rating system, 26 (96.3%) of the 27 replicate respondents had “good” or “excellent” consistency. Pearson’s correlation coefficients are sensitive to outliers, and the interview with poor consistency (0.59) was determined to have had one outlier, which likely affected the score. 

An integrated database was created that included data from a total of 435 completed surveys: 289 from the 2016–2017 BHC survey (those from the impact and buffer areas) and 146 from the 2018 GHIB survey. New variables were created and included in the database to reflect: the year in which the survey was conducted (2016–2018); whether the respondent lived in the impact area or the buffer area; the version of the questionnaire used; whether the survey respondent was part of the replicate sample (*n* = 27); and whether the respondent lived within 300, 500, or 1000 feet of a heavily trafficked roadway (I-75), or one of the trucking routes through the neighborhoods. To facilitate comparisons across surveys, numeric designations for all survey items included in both surveys were converted to the 2018 questionnaire numbering system in an integrated codebook. Based on the demographic findings and census data from the 2012–2016 ACS, survey weights were constructed that allowed survey results to be extended to the GHIB study area population.

## 4. Results

The HIA encompasses a collection of current population data and estimates of potential health impacts, along with 15 pages of recommendations for ways to mitigate potential adverse impacts and amplify health benefits. Here, we share findings, including select demographic and health characteristics, neighborhood perceptions, plans to move, and top neighborhood concerns related to the GHIB, as well as select recommendations in the HIA that these specific findings inform. Many additional findings are reported in the full HIA [8], as well as 10 recommendations that aim to do one of the following:

(1) Reduce emissions of air pollutants or noise associated with the new GHIB;
(2) Reduce the exposure of residents to air pollutants or noise emitted as a result of GHIB activity;
(3) Reduce adverse health effects among residents whose health is impacted by air pollutants or noise in the GHIB area.

### 4.1. Demographic & Health Characteristics 

Table 1 shows the demographic characteristics of the survey respondents (2016–2018), compared with census data from relevant census block groups drawn from the 2012–2016 American Community Survey (ACS) [28]. The full sample includes 435 adult survey respondents (age 18 and older), who reported on the health of 1629 household members of all ages in total. Age and education distributions of survey respondents were similar to ACS values for the GHIB area.

**Figure 8:**
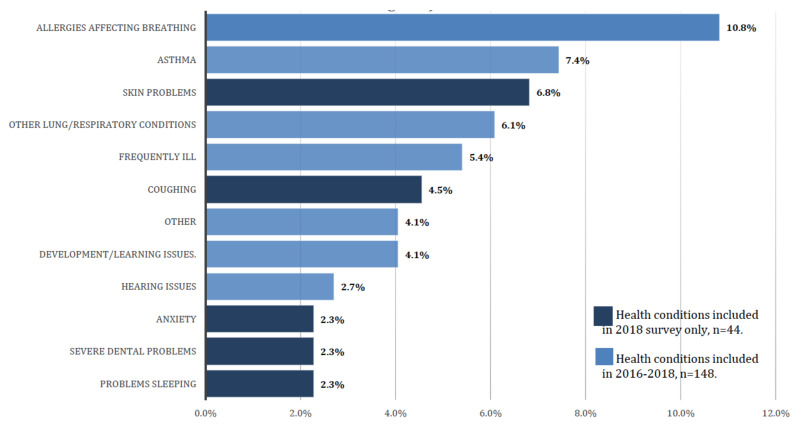
Health issues reported for children under the age of 5 years old (*n* = 148, unweighted).

**Figure 9:**
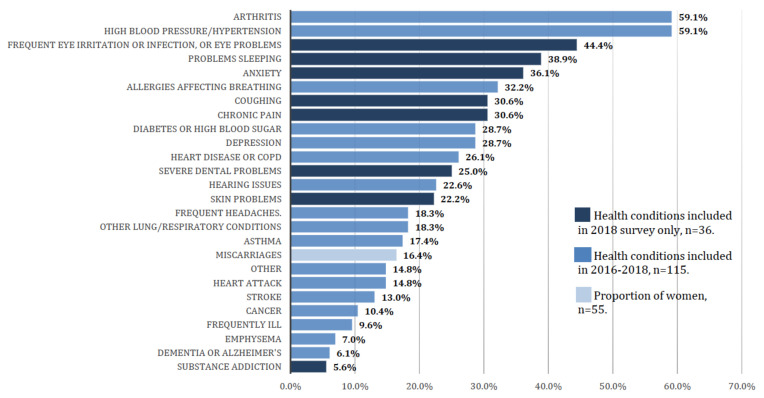
Health issues reported for adults aged 65 years and older (*n* = 115, unweighted).

Survey respondents were asked to report on the health of other members of their household, and the diverse and extensive health concerns informed a recommendation to expand access to health care, by assuring access to health insurance, neighborhood-based mental and physical health care services, including basic screening, and addressing transportation issues. Here, we report results on health status, inclusive of all household members (*n* = 1629) for children 5 years and under (Figure 8) and adults 65 years and older (Figure 9). Of the 148 children under the age of five, on whom data was obtained, 10.8% were reported to have allergies affecting breathing, followed by asthma (7.4%), skin problems (6.8%), and other lung/respiratory conditions (6.1%). Among those aged 65 and older, the most frequently reported health issues were arthritis (59.1%), and high blood pressure or hypertension (59.1%). 

We also share key findings related to asthma (Table 2) that informed our recommendations that: (1) spatial buffers of at least 500 feet should be placed between heavily trafficked roadways and land uses with sensitive populations, such as schools, hospitals, clinics, and nursing homes; and (2) household relocation opportunities are expanded to a minimum of 500 feet. One in five (19.0%) children aged 5–18 living within 500 feet of I-75 or trucking routes were reported to have asthma. This is higher than the proportion reported for children in the same age group living more than 500 feet from these truck routes and the I-75 freeway (13.4%). Among those under 18 years of age, there was a statistically significant difference in percent asthma (*p* < 0.10) and allergies affected breathing or asthma (*p* < 0.05) reported among those living 500 feet or less, compared to those living more than 500 feet from heavily trafficked roadways. Health status is reported stratified by age, so the percentages reported are unweighted. The full HIA report presents comprehensive findings for most common health outcomes across all age strata, as well as birth outcomes. 

### 4.2. Neighborhood Perceptions and Plans to Move 

Members of the CBC and CAG wanted the HIA to document neighborhood perceptions and residents’ plans to stay or move, in order to frame discussions around community benefits and inform HIA recommendations accordingly. Table 3 shows the percent of respondents who agreed or strongly agreed with a series of five items asking each respondent about their sense of connection to their neighborhood. Both unweighted and weighted results are presented. More than four out of five respondents agreed or strongly agreed that people in their neighborhood generally know each other, and that they feel at home in their neighborhood. Seven in 10 respondents indicated that they thought the neighborhood was a good place for them to live and that people in the neighborhood share the same values, with just under seven in 10 expecting to live in the neighborhood for a long time. More than six in 10 indicated that it was important to them to live in this particular neighborhood. The results shown in Table 4 compare responses to this question for those living in the impact area and those living in the buffer area north of the bridge. The residents of the impact area were more likely to indicate that they planned to move within one year, or within the next 1–5 years, compared to those living in the buffer area north of I-75, with those living in the buffer area more likely to indicate that they were not planning to move. Differences were statistically significant (*p* = 0.01).

Respondents were asked to respond to sets of items asking about problems in their neighborhood, and these directly informed several of the ten HIA recommendations. As shown in Table 5, in 2018, four out of five household respondents reported that rats were very much a concern (81.6%), and three out of four (76.1%) indicated that clogged sewers or standing water in streets were very much a concern. With over half of respondents expressing concern over truck traffic as a safety concern, the HIA includes a recommendation that trucking routes that have the least impact on residential neighborhoods and areas with sensitive populations be established and enforced. 

## 5. Discussion

The results reported here show how community-led efforts focused on understanding and addressing the impacts of major transportation infrastructure projects on a local level led to an increased knowledge of the potential health impacts of that infrastructure, and specific strategies for reducing potential adverse health impacts. Selected results from the HIA that were attained through the organizing efforts of the CBC suggest that children under 5 years old and adults age 65 and older living in the GHIB reported respiratory and, amongst the older population, cardiopulmonary challenges, which are associated with exposure to airborne particulate matter [20,21,22]. Those aged 5–18 years old who lived within 500 feet of heavily trafficked roadways in the area reported higher levels of asthma and allergies related to breathing than did those who lived more than 500 feet from those roadways. Notably, both groups have higher self-reported rates than estimated national asthma prevalence. These results reflect baseline conditions, prior to the construction of the GHIB. Combined with results from other studies [11] demonstrating near roadway health effects, these findings suggest particular cause for concern for residents of the area as bridge construction and operations begin. These findings highlight the importance of recommendations for reducing exposure of residents who live near the bridge and local trucking routes, as traffic and emissions increase in the area with bridge-related activities.

A second set of findings reported above is related to residents’ sense of connectedness to their neighborhoods and the likelihood that they will move away from the neighborhood within 1–5 years. In general, at baseline, residents of the area to be impacted by the GHIB report feeling positively about their neighborhood, with more than four in five indicating that they felt at home in their neighborhood, and that people in the neighborhood knew each other. Roughly seven in 10 reported that the neighborhood was a good place for them to live, that they shared values with their neighbors, and expected to live in their neighborhood a long time. A second set of questions asking about specific plans to move suggested some differences between residents of the immediate GHIB impact area and residents of the larger surrounding neighborhood. Specifically, those who currently lived in or immediately adjacent to the footprint of the new bridge were significantly more likely to indicate that they planned to move within the next 5 years, compared to those living in the buffer area. Together, these results suggest that, despite the strong sense of community that currently exists in the area, those living near the GHIB footprint are likely to move away. Displacement—whether voluntary or involuntary—has been shown to have adverse health impacts [29]. Recognizing these health impacts and identifying recommendations for reducing adverse health effects, offers another opportunity to inform specific recommendations for reducing adverse health impacts associated with the bridge. 

The third set of findings described above focuses on specific neighborhood concerns identified by residents of the area to be affected by GHIB construction and operations. The 2018 survey was conducted as construction on the bridge was beginning. Hence, concerns raised likely reflected some related to the construction process and its impacts on residents, as well as longer term concerns as the bridge will become operational. As noted above, concern about rats was high at a time when sewer rats were displaced when underground sewers were dug up and disrupted in the construction process. Other specific concerns had to do with construction equipment and trucks in the neighborhood, disrupting traffic flow and causing flooding as storm sewers were disrupted due to construction. Residents noted multiple concerns about air quality, including road dust from daily traffic and construction equipment, fumes and emissions affecting outdoor air quality, and indoor air quality, as those emissions made their way into homes. Finally, concerns about vibrations from truck traffic, impacting the structural integrity of homes, were articulated by more than 50% of survey participants. The HIA developed specific recommendations related to each of these issues and concerns, in order to address adverse health and economic impacts for residents of the immediate area. 

Together, the results from the HIA survey both documented baseline health conditions in the community in which the GHIB is being constructed, and provided a basis for specific recommendations to reduce those adverse health impacts. The fact that the HIA was conducted was the result of years of organizing and engagement on the part of the SW Detroit CBC, and speaks to the role of local organizing to provide information about the local implications of transportation development. Once completed, the results from that study and the specific recommendations for strategic actions to reduce adverse health impacts, guided the continued work of the SW Detroit CBC to protect the health of the area’s residents. Below, we discuss the broader implications of this case study of community organizing to understand and address health impacts of transportation infrastructure. 

Implications for understanding health impacts of decisions about transportation infrastructure. Federal and state decisions about transportation infrastructure have long contributed to social, economic, and health inequities in the U.S. The Federal Highway Act of 1956 is credited with connecting rural and urban communities with increased economic opportunities for many, but the 48,000 miles of highway built nationwide also led to the loss of many cities’ tax bases, and the decimation of many communities of color in the name of urban renewal [30,31]. Today, major U.S. international freight gateways are a major source of air pollution and are frequently located in communities of color and low-income communities [32,33], as seen in the case of the GHIB. Some states are beginning to assess cumulative impacts in permit decisions for industrial pollution sources [34,35], but, currently, there are no federal standards for identifying the disparate or cumulative impacts or mitigations related to transportation infrastructure. While it is important to consider regional impacts, processes are needed to ensure consideration of potential localized health impacts of new infrastructure, particularly for communities residing proximal to other emissions sources and with other social vulnerabilities. The case of the GHIB in Delray and nearby Southwest Detroit illustrates how a community mobilized to advocate for an HIA as part of community benefits, and provides one approach to consider the ways health and environmental justice may be brought into infrastructure deliberations. 

Although there are no universally established HIA guidelines [36], a ‘traditional’ HIA might have been done earlier, to inform the GHIB’s location and design. The City-State Agreement that made this HIA possible was signed after primary development decisions and permitting were approved. The Agreement specifies that the HIA occur in three phases (i.e., pre-construction, construction, and operation). The case of the GHIB, particularly the resulting evidence-based recommendations, illustrates how many decisions there are within a major infrastructure decision that may impact the exposure pathways, magnitude, and frequency experienced by nearby residents. This HIA will also include two additional waves of data collection—during construction and during operation—that may allow for evaluation of the potential impacts of the bridge as it is built and begins operating. This allows for the HIA to understand changing contexts, as well. For example, in 2018, residents reported concerns with rats and sewer infrastructure that were minimally referenced in the BHC survey, as construction had since begun to affect their community’s quality of life in new ways. This suggests there are benefits from a phased HIA, which provides an opportunity to adapt and make relevant recommendations over time. 

Implications for quality of life in communities disproportionately impacted. As the GHIB is a multi-billion-dollar international development, intended to be profitable for decades to come, residents and allies believed initial environmental protections were insufficient and out of proportion with the scope of the project. It is often difficult to secure protections without proof of impacts, and epidemiological approaches are often limited in their ability to show causation between environmental factors and health outcomes [37]. Additionally, many social and health indicators, such as personal safety and residents’ attachment to place and neighbors [38], are not considered formally during the environmental planning processes. In addition to more than a decade of community mobilization, the preliminary findings from the 2016–2017 BHC survey were a catalyst that helped decision-makers to see the need for a formal HIA. Findings related to neighborhood perceptions and concerns and intentions to stay or relocate, as well as open-ended responses reported in the HIA, help to humanize infrastructure decisions for decision-makers with a broad understanding of community health and well-being. Our survey findings were consistent with a substantial body of evidence suggesting that living near heavily trafficked roadways is associated with higher rates of asthma [11], illustrating how key scholarly findings manifest locally in people’s lives. An EIS that considers regional rather than local impacts, and an eminent domain that compensates only those living in the immediate footprint of a project, does not fully consider or address the impacts on quality of life for the many remaining residents living alongside the new infrastructure. 

GHIB HIA recommendations, and the findings underlying them, may be useful in informing community benefits discussions underway in real time. For example, the home swap program has considered neighborhood conditions (e.g., distance to heavily trafficked roadways) when identifying potential new homes for displaced Delray residents. Among the many GHIB HIA findings, we report that the proportion of self-reported asthma was greater among children living within 500 feet of I-75 or trucking routes through the GHIB area—16.3%, compared with the 11.6% of those living more than 500 feet away. Residents, particularly older adults, report a host of existing health issues, such as hypertension, anxiety, sleeping problems, hearing issues, and frequent headaches. Residents voiced multiple concerns about the potential social and economic implications of the bridge, including home damage due to vibrations from heavy trucks, loss of home value, exposure to construction noise, increased truck traffic through residential areas, and increased exposure to dust and air pollutants. These findings suggest that the following strategies are among those that can be used to reduce adverse health impacts of GHIB construction and operations moving forward:

- Expanding the optional home swap program to include all residents living within 500 feet of the new bridge;
- Expanding the number of homes eligible for windows and indoor air filters to reduce indoor air pollutants;
- Assuring that entrance and exit ramps and truck routes avoid residential neighborhoods;
- And increasing enforcement of existing idling policies and truck routes.

Implications for understanding the potential for community benefits to protect health. The GHIB case study shows that community benefits can take many forms. Communities often advocate for benefits that are basic amenities to counteract the burden of hosting a polluting site. In and of themselves, HIAs should not be considered a ‘benefit.’ However, funding for public health to achieve its core functions of assessment, policy development, and assurance [39] is limited, even though efforts to address environmental justice do fall within public health’s purview, if done with communities, to address health inequities [40]. State environmental agencies are often limited in their ability to extensively monitor air toxins and mobile source emissions [41]. Many frontline communities experiencing existing cumulative impacts advocate for health studies and, in the case of the GHIB, the CBC advocated that this be part of the community benefits for residents who were concerned about the additional environmental exposures associated with its development and operation. Since impacts of the development can be anticipated but not fully be known in advance, the HIA and air monitoring can demonstrate impacts later, and lay the basis for potential additional mitigation. HIAs can be powerful tools to assess cumulative impacts when traditional risk assessment methods are limited [27].

This case study also highlights the complexity of addressing the potential effects of major infrastructure on health, given the decision-making role of multiple agencies, sectors, and levels of government. Over more than a decade, the Delray and SW Detroit community were tasked with educating three mayors, three Governors, over 20 City Council members, and various agency staff, as well as Canadian elected officials, of community concerns. Across the U.S., there are similar frontline communities where legacy industrial zoning, with a lack of environmental protections, contributes to the ongoing citation of polluting facilities. Particularly in these vulnerable areas, advocacy is needed at all levels to improve public health. Consideration of the cumulative impacts and community benefits could be better integrated into state-defined air pollution permitting processes. At the local level, reconsideration of zoning in ways that considers public health may help to address cumulative impacts [42], or cumulative impacts ordinances may be implemented as seen in Newark, NJ [43]. In 2016, the City of Detroit passed a Community Benefits Ordinance [44] that was predicated on the work of the CBC, although more limited, as well as community organizing initiatives related to other developments in the city including Little Caesars Arena and the Herman Kiefer redevelopment. 

A role for HIAs in centering community in land use and infrastructure development. Public participation (sometimes known as public involvement or engagement) is mandated in some agency-led land use planning by many federal laws, but this cannot guarantee environmental justice without concerted efforts to ensure that participation is anti-racist and meaningful [45]. As Woolcock [46] notes, “Many development agencies and governments now seek to engage directly with local communities, whether as a means to the realization of more familiar goals (infrastructure, healthcare, education) or as an end in itself (promoting greater inclusion, participation, well-being).” Yet, the latter is often at odds with economic forces and project timelines in which developers have different end goals than local community residents and businesses [46]. Integrated into community benefits agreements or ordinances, HIAs may be tools for agency staff to engage and identify community concerns, in a way that puts the most heavily impacted community at the center of complex infrastructure decisions to better understand vulnerability and impacts, as well as potential remedies. Many more examples of how to ensure true community input in the HIA process are needed, though [47,48]. 

This case study highlights that community must be centered. This can be done by building on local assets, intentionally designing structures (e.g., the CAG) and points for input, integrating input into HIA design and execution, grounding partnerships in community-based participatory research principles [49], and growing agency capacity for approaches that sincerely promote equitable processes and outcomes. Community and academic partners were contracted by the Detroit Health Department (DHD) to complete the HIA. Strong relationships between academic and community partners who had been working together previously created space for the government agency to engage with community from a place of trust, at a time when the DHD was rebuilding after Detroit’s bankruptcy. By engaging the CBC and longtime partners, the HIA process built on existing community-initiated research (i.e., the BHC survey) in rigorous, validated ways and existing community bodies, such as the CAG and the CBC’s Resident Engagement Committee, to ensure the methods and interpretation of findings were locally relevant. Alongside researchers, the CBC fully influenced design, data collection, interpretation of results, and generation of evidence-based recommendations. These partners were able to build skills and knowledge locally and within the DHD, as this was the first HIA some agency staff had ever overseen. 

Finally, while there is no gold standard for how to conduct an HIA, we should be critical of methodology underlying HIAs, including the GHIB HIA. Our survey is based on a census approach, and relies on self-reported data. Although this approach may underestimate undiagnosed cases of some illnesses, such as hypertension or asthma, it was vetted for validity through scholarly and community perspectives. Given the cross-sectional nature of our survey data, we are limited in our ability to fully eliminate single-source biases or draw causal inferences. Future waves of the HIA may be able to provide some longitudinal insights to health conditions and concerns in the community. Additionally, neighborhood changes and the ongoing implementation of the City-State Agreement must be considered in interpretation of findings in this three-phase HIA. For example, those living in the impact area were more likely to have been interviewed in 2016–2017, while those living in the buffer area were more likely to have been interviewed in 2018, and those temporal differences may have influenced differences in reporting between the groups. In addition, those living in the impact area are closer to the footprint of the new bridge and are more likely to be eligible for financial support for moving, compared with those living in the buffer area, potentially contributing to greater likelihood of indicating plans to move. It is also likely that response distributions to this item may change as bridge construction begins, as residents may feel more motivated to move once they experience impacts of bridge construction and operation, and will likely affect different areas at different points in time.

## 6. Conclusions

Under the Clean Air Act, state monitoring, permitting, and enforcement do not address cumulative impacts related to air pollution in most states, including Michigan. Nor are there codified mechanisms for addressing the impact of major infrastructure developments on community quality of life. Without these public health protections, the strategic use of HIAs to monitor and inform efforts to mitigate potential adverse health effects of land use and infrastructure decisions offers critical opportunities to promote environmental justice in disproportionately impacted communities. Community benefits awarded in Detroit included resources to document baseline social and health indicators, inform specific recommendations for actions to mitigate potential adverse impacts, monitor air quality, and track the GHIB’s human impact over time. While HIAs are typically designed to inform decisions prior to being finalized, in the instance of the GHIB, the decision to build an international border crossing had already been made by federal, state, provincial, and local governments. However, there remained opportunities to inform multiple decisions related to the process of building and operating the GHIB: design, truck routes, workforce opportunities, and policies or programs to protect the fence line community. In this instance, the CBC advocated to keep the community at the center to inform those decisions to assure that the HIA reflects the experience of residents. Finally, this case study provides lessons learned for community, academic, and government partners conducting HIAs during building and operation of major infrastructure. Based on the experience presented here, effective community mobilization was essential to creating the opportunity to conduct an HIA to understand likely impacts on quality of life, and the application of findings to inform decision making in order to protect residents. Together, community mobilization and advocacy, scientific evidence regarding implications for quality of life, and strategic recommendations for health promotive actions are powerful and necessary components of efforts to achieve environmental justice. 

## Figures and Tables

**Table 1:** Demographic characteristics of survey respondents, household members, and GHIB census block group population.

Demographic Variables		Survey Respondents (2016, 2018) (*n* = 435) (A)	American Community Survey ≥ 18 Years Old (*n* = 11,320) * (B)	*p*-Value A vs. B	Survey Household Members (*n* = 1629) (C)	American Community Survey All Household Residents (*n* = 16,382) (D)	*p*-Value C vs. D	*p*-Value A vs. D
Age (in years)	≤4			0.4343	9.1	9.9	0.084	
	5–17				27.9	21		
	18–64	85.9	87.2		56	60.3		
	≥65	14.1	12.8		7.1	8.8		
Gender **	Female	68.1	50.5	<0.001	n.a.	50.7		
	Male	32.0	49.5			49.3		
Education	<High School Graduation	48.7	n.a.		n.a.	46.2		0.464
	High School Graduation	28.9				30.6		
	>High School Graduation	22.4				23.3		

* American Community Survey data for education includes those 25 years and old, thus this comparison includes only survey respondents 25 years and older (*n* = 415) ** Gender is available for survey respondents only in 2018, thus not included in statistical comparison for all household members.

**Table 2:** Percent reported asthma by age group among those living 500 feet or less and more than 500 feet from heavily trafficked roadways (unweighted data, 2016–2018, *n* = 1690).

Self-Reported Asthma or Allergies Affecting Breathing or Asthma	Age Group	GHIB Area		GHIB Area
		≤500 Feet	>500 Feet	All
Asthma	<18 years *	16.3	11.6	13.8
	Under 5 years	9.8	4.6	7.4
	5–17 years	19.0	13.4	15.9
	18–40 years	9.1	6.6	7.7
	41–64 years	14.4	13.6	13.9
	≥65	24.4	12.9	17.4
Allergies affecting breathing or asthma	<18 years **	28.7	20.4	24.3
	Under 5 years	18.3	10.6	14.9
	5–17 years **	33.0	22.9	27.4
	18–40 years	25.5	20.9	22.9
	41–64 years	34.8	29.7	31.9
	≥65	42.2	32.9	36.5

* *p* < 0.01 ** *p* < 0.05.

**Table 3:** Percentage of respondents who agree or strongly agree with statements about their neighborhood.

Perceptions of Neighborhood	*n* *	% Agree or Strongly Agree Unweighted Weighted	
I think this neighborhood is a good place for me to live.	431	70.3	70.5
People in this neighborhood share the same values.	417	69.5	69.8
I feel at home in this neighborhood.	431	85.2	85.6
It is very important to me to live in this particular neighborhood.	433	63.3	64.0
I expect to live in this neighborhood for a long time.	424	68.6	69.6
People in this neighborhood generally know each other.	429	81.1	81.9

* n’s vary due to missing data.

**Table 4:** Intention to move by residence in impact or buffer areas of GHIB (*n* = 435, weighted percent).

Plan to Move	% Living in Impact Area (*n* = 224)	% Living in Buffer Area (*n* = 211)	Statistical Test of Difference *
Within one year	8.6	5.3	0.01
Between 1–5 years	17.7	10.4	
More than 5 years	5.9	4.7	
Not planning to move	57.8	73.6	

* *p*-value corresponds to test of independent proportions—Chi-square test.

**Table 5:** Neighborhood concerns reported by survey respondents, 2018 (*n* = 146, weighted percent).

Neighborhood Concerns	Very Much a Concern (%)	Somewhat of a Concern (%)	Not a Concern at all (%)
Rats	81.6	9.2	9.2
Traffic congestion making it hard to get places	76.1	8.8	15.9
Clogged sewers or standing water in streets	75.3	11.6	13.1
Outdoor air quality, such as emissions from trucks industry, fumes or odors	66.7	18.8	14.6
Vibration from trucks or construction activity damaging property	60.9	18.4	20.7
Vacant houses	60.4	16.2	23.3
Road dust	60.2	20.0	19.9
Loss of property value	56.7	21.3	22.0
Crime	55.0	23.9	21.1
Truck traffic on residential streets affecting safety	54.8	16.1	29.1
Noise during sleeping hours	47.1	20.3	32.6
Indoor air quality, such as fumes and dust inside the house	41.4	25.1	33.5
Noise during the day	40.9	29.8	29.3
Many residents moving away	23.8	35.6	40.6
